# Primary hyperparathyroidism due to a giant parathyroid adenoma presenting with pathological fractures and multiple brown tumors

**DOI:** 10.1530/EDM-24-0054

**Published:** 2024-12-19

**Authors:** Jassy Meng, Wedyan M Aboznadah, Marc Pusztaszeri, Vincent Larouche

**Affiliations:** ^1^ General Internal Medicine Fellowship Program, McGill University, Montreal, Quebec, Canada; ^2^ Adult Endocrinology and Metabolism Fellowship Program, McGill University, Montreal, Quebec, Canada; ^3^ Department of Medicine, King Abdulaziz University, Jeddah, Saudi Arabia; ^4^ Department of Pathology, Jewish General Hospital, McGill University, Montreal, Quebec, Canada; ^5^ Division of Endocrinology and Metabolism, Jewish General Hospital, Department of Medicine, McGill University, Montreal, Quebec, Canada

**Keywords:** calcium, bone, rare diseases/syndromes

## Abstract

**Summary:**

Primary hyperparathyroidism (PHPT) is a disorder in which excessive parathyroid hormone (PTH) is secreted from the parathyroid glands. The cause of PHPT is most commonly parathyroid lesions such as parathyroid adenoma. The clinical manifestations of PHPT include hypercalcemia, nephrolithiasis, bone disease and rarely pathological fractures and brown tumors, which arise within the foci of osteitis fibrosa. Brown tumors are benign intraosseous tumors that occur because of excessive osteoclast activity. Because of advances in medical care, early diagnosis and treatment have meant that diagnosing PHPT in the setting of multiple brown tumors is particularly rare. Here, we present a case of a young man with prolonged PHPT from a giant parathyroid adenoma with multiple brown tumors causing pathological fractures. Definitive treatment of PHPT is parathyroidectomy with particular attention to the risks for hungry bone syndrome (HBS) postoperatively.

**Learning points:**

- Pathological fractures from brown tumors are a rare but significant concern in prolonged PHPT, and a multidisciplinary approach is required including orthopedic surgery, otolaryngology and endocrinology.
- It is important to assess PTH and calcium in the context of hypercalcemia with bone lesions to avoid potential diagnostic delays.
- Postoperative parathyroidectomy patients with large parathyroid adenomas, elevated alkaline phosphatase, elevated PTH and the presence of brown tumors are at particularly high risk for HBS.
- Very high levels of PTH, calcium, alkaline phosphatase and multiple brown tumors should raise concern for a potential case of parathyroid carcinoma.
- Indications for genetic testing for inheritable parathyroid disease include patients younger than 30 years old, those with multigland disease, those with a family history of hypercalcemia or syndromic disease and those with atypical parathyroid adenoma and parathyroid carcinoma.

## Background

Primary hyperparathyroidism (PHPT) is a disorder in which excessive parathyroid hormone (PTH) is secreted from one or more of the parathyroid glands. The cause of PHPT is most commonly parathyroid adenoma (85%), followed by parathyroid hyperplasia (15%) and parathyroid carcinoma (<1%) (1). Rare genetic disorders can be associated with PHPT, such as multiple endocrine neoplasia type 1 and type 4, hyperparathyroidism–jaw tumor syndrome, familial hypocalciuric hypercalcemia and familial isolated hyperparathyroidism.

The clinical manifestations of PHPT include hypercalcemia, nephrolithiasis, bone disease and rarely pathological fractures and brown tumors. Brown tumors arise within foci of osteitis fibrosa and are benign intraosseous tumors that occur because of excessive osteoclast activity, resulting in increased bone resorption and bone remodeling. They are called brown tumors because of the histopathological brown coloration due to hemosiderin deposition. They can be found in any part of the skeleton but are more commonly found in the skull, pelvic bones, long bones and ribs. Brown tumors are usually solitary lesions and rarely present as multiple lesions (2, 3, 4).

Because of advances in medical care, early diagnosis and treatment of hyperparathyroidism have become commonplace. Therefore, diagnosing PHPT in the setting of multiple brown tumors is exceedingly rare in this part of the world. However, brown tumors are seen with more frequency in parts of the world where vitamin D deficiency is an endemic, such as India, Pakistan, China and the Middle East (2, 3, 4). Incidences of multiple pathological fractures due to several brown tumors are even rarer.

Here, we present a unique case of a young man diagnosed with severe PHPT with pathological fractures of the long bones and multiple brown tumors from a giant parathyroid adenoma, who also developed hungry bone syndrome (HBS) in the postoperative period.

## Case presentation

A 38-year-old man with no past medical history presented to our emergency room (ER) complaining of acute pain on chronic right-sided hip with inability to ambulate after coughing. This was accompanied by polyuria and polydipsia. He was found to have a pathological hip fracture on X-ray and multiple deep vein thrombosis (DVT) in the same leg. The patient had a negative family history of calcium metabolism disorders. Prior to his presentation, he had persistent right-sided hip pain the last 8 months, which did not respond to physiotherapy or over-the-counter pain medications. A month prior to this ER visit, he was seen by a physical medicine and rehabilitation physician as an outpatient, who ordered an MRI that showed diffuse lytic bone lesions. For unclear reasons, the patient did not present to the ER as advised at that time.

## Investigation

**Figure 1:**
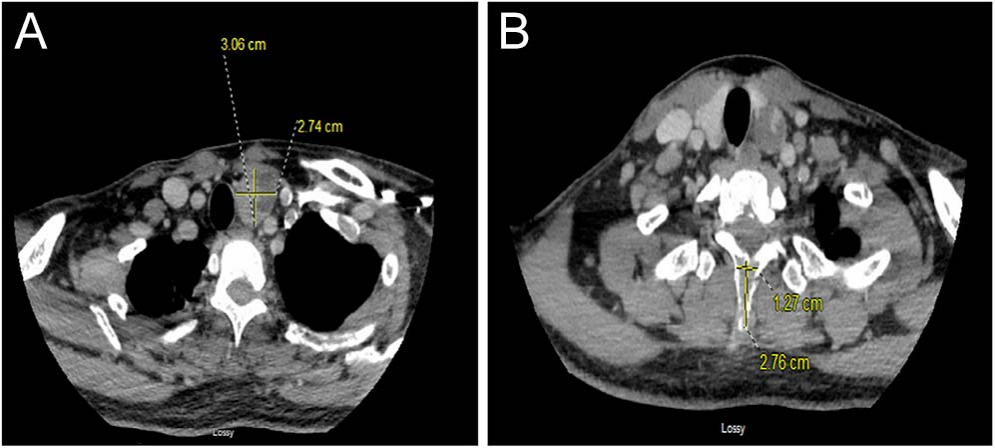
(A) CT scan of the neck: there is a mixed solid and cystic mass along the inferior margin of the left thyroid lobe measuring 2.7 × 3.0 × 3.4 cm (preoperative image). (B) An oval lucent lesion is noted in the anterior body of C5 measuring 0.9 cm. There is an expansile lytic lesion in the transverse process of T1 measuring approximately 1.2 × 2.8 cm (preoperative image).

**Figure 2:**
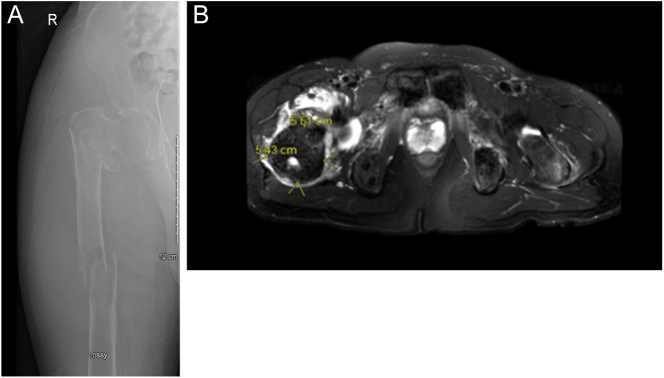
(A) Femoral X-ray: right proximal femoral diaphyseal pathologic obliquely oriented fracture through a suspicious lytic lesion measuring at least 7.0 × 3.5 cm. Fracture has resulted in mild medial and posterior displacement as well as minor apex medial/posterior angulation. Affected intertrochanteric right proximal femoral fracture with abnormal lucency in the right greater trochanter compatible with a pathological fracture. Extreme cortical thinning is shown in this X-ray, which is unique and specific to PTH-mediated bone loss. (B) MRI of the hips and femur: the largest mass lesion is within the right intertrochanteric region with associated pathological fracture and its surroundings (preoperative image).

**Figure 3:**
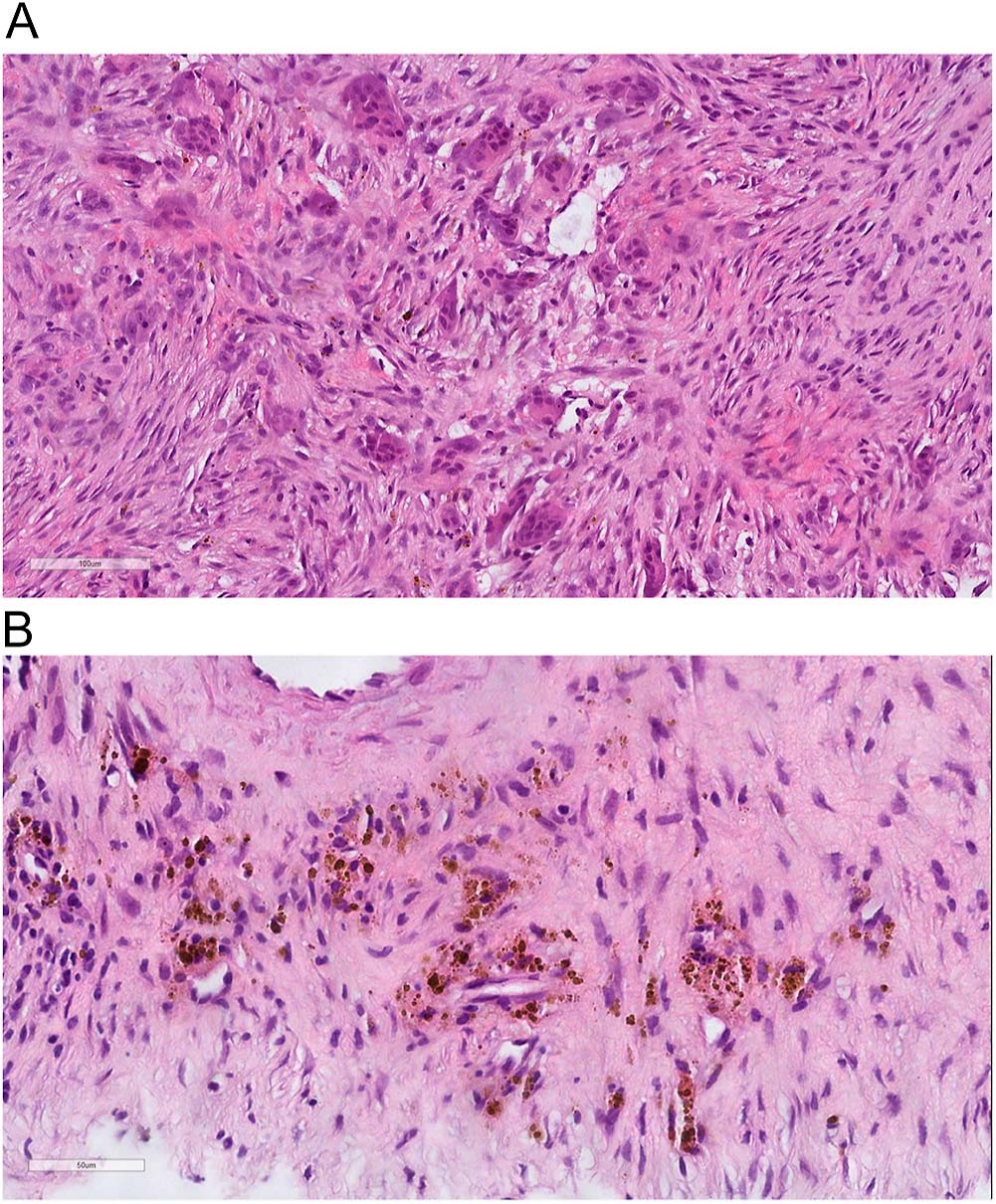
(A) Brown tumor with clusters of osteoclast-like multinucleated giant cells in a fibroblastic stroma. (B) Brown tumor associated with abundant brown pigment (hemosiderin) deposits.

He was found to have hypercalcemia with an adjusted calcium of 3.54 mmol/L (normal range: 2.12–2.62 mmol/L) and an acute kidney injury with a creatinine of 113 μmol/L (patient’s baseline: 60 μmol/L) (Table 1). A CT scan of the chest and abdomen showed nephrolithiasis and diffuse lytic bone lesions but no obvious primary malignancy. An MRI of the pelvis confirmed a right-sided intertrochanteric fracture with a large-mass lesion at the site of the fracture along with more than 20 numerous lesions involving the lumbar spine, pelvis and bilateral femurs. The patient was then diagnosed with PHPT with an elevated PTH of 1089 ng/L (normal range: 10–70 ng/L). Subsequently, a CT scan of the neck was ordered, which showed a mixed solid/cystic mass along the inferior margin of the left thyroid lobe measuring 2.7 × 3.0 × 3.4 cm (Fig. 1A and B). Similarly, a preoperative ultrasound performed by the ears, nose, throat ENT surgeon (otolaryngologist) confirmed a large 3 cm left inferior parathyroid lesion with a mixed cystic and hypoechoic solid component. Unfortunately, while waiting for surgical repair of his right intertrochanteric fracture, he suffered a second proximal femoral diaphyseal fracture in the same leg (Fig. 2A and B). He was seen by orthopedic surgery and underwent surgical repair with a bone biopsy taken intraoperatively. Pathology was consistent with a brown tumor, demonstrating clusters of osteoclast-like multinucleated giant cells in a fibroblastic stroma, associated with abundant brown pigment (hemosiderin) deposits (Fig. 3A and B). Because part of the differential diagnosis included parathyroid carcinoma and hyperparathyroidism–jaw tumor syndrome, genetic testing for *MEN1*, *CDC73*, *AP2S1*, *CASR*, *CDKN1B*, *GNA11* and *RET* was ordered and resulted negative.

**Table 1:** Patient’s biochemical values at diagnosis and postoperation. Values outside the reference range are presented in bold.

Variables	Patient’s values			Reference range
	At diagnosis	8 months postoperation	12 months postoperation	
Calcium, mmol/L	**3.56**	**2.08**	2.36	2.12–2.62
Albumin, g/L	42	46	47	35–51
Albumin-adjusted Ca, mmol/L	**3.54**	**2.08**	2.36	2.12–2.62
PHT, ng/L	**1089**	**143**	**126**	10–70
Alkaline phosphatase, U/L	**248**	NA	88	40–125
Phosphorus, mmol/L	**0.66**	0.89	1.20	0.70–1.45
Magnesium, mmol/L	0.80	0.80	0.81	0.70–1.23
25 (OH) vitamin D, nmol/L	**16**	**73**	**56**	Insufficient: 25–75
1.25 vitamin D, pmol/L	**86**	NA	NA	90–174
Creatinine, μmol/L	**113**	95	99	55–110
GFR, ml/m/1.7sm	>60	>60	>60	60–1000

GFR, glomerular filtration rate; PHT, parathyroid hormone.

**Figure 4:**
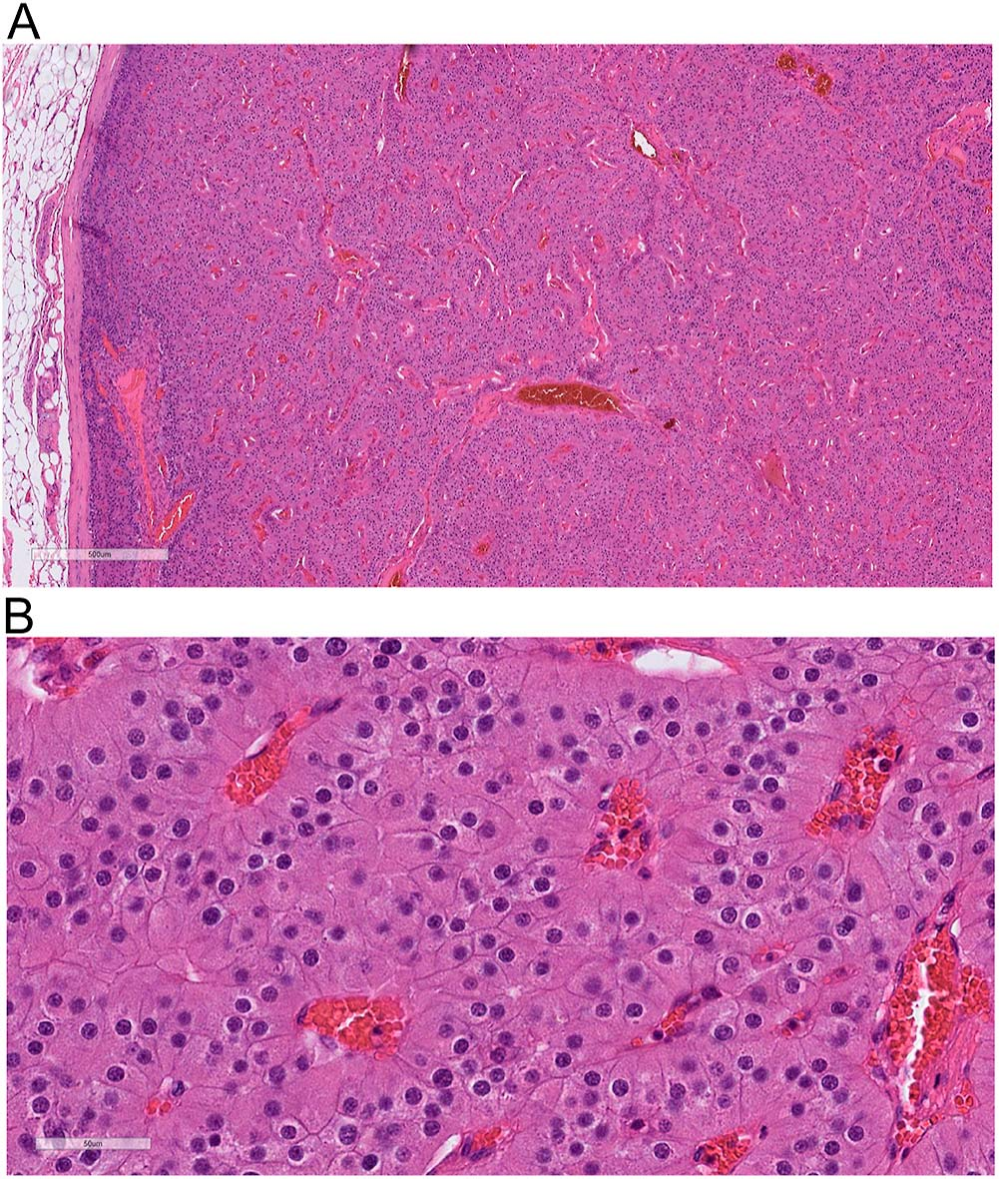
(A) Parathyroid adenoma, oncocytic type. At low magnification, the tumor is well demarcated by a fibrous capsule with no signs of invasion. (B) At high magnification, it consists of solid clusters of oncocytic cells with no significant nuclear atypia.

The patient then underwent a Tc-99m sestamibi scan of the parathyroid glands, which redemonstrated the large left parathyroid lesion Perrier type E. The Perrier classification is used by radiology and nuclear medicine physicians to provide a means of consistent communication about parathyroid adenoma location, which may be beneficial for surgical planning as well as operative and pathology reporting. A type E lesion refers to an inferior gland close to the inferior pole of the thyroid parenchyma, lying in the lateral plane with the inferior thyroid gland and anterior half of the trachea. He was then referred to otolaryngology and underwent an en bloc resection of the parathyroid gland with a left hemithyroidectomy and central neck dissection within 2 weeks of the initial diagnosis. The histopathological examination of the excised mass revealed a parathyroid tumor that was totally encapsulated with no evidence of invasion (capsular, vascular or perineural), not meeting criteria for parathyroid carcinoma but was most consistent with an adenoma measuring 3.5 × 3.3 × 3.0 cm, with a weight of 33 g, consistent with a giant parathyroid adenoma (Fig. 4A and B).

## Treatment

On initial presentation to the ER, the patient was treated with aggressive intravenous hydration (200 cc/h of normal saline, NaCl: 0.9%) over 48 h and calcitonin 400 international units. As preferred by the orthopedics service, a bisphosphonate was not given immediately for his pathological fracture as its administration can impair bone healing in the postoperative setting. Although most studies show no difference in fracture healing when bisphosphonates are administered in the immediate postoperative period, this study has shown an increased risk of nonunion with bisphosphonates (5). Considering this uncertainty, combined with the rapid normalization of calcium with intravenous fluids and calcitonin only and his mild acute kidney injury, a decision was made to not administer bisphosphonates initially.

He remained largely asymptomatic despite his moderate hypercalcemia. He was also found to be vitamin D-deficient with a level of 16 nmol/L (insufficient: 25–75 nmol/L) and was prescribed vitamin D 10,000 units weekly (Table 1). A meta-analysis showed that vitamin D replacement in subjects with PHPT and coexistent vitamin D deficiency increases 25 (OH) D levels and reduces serum PTH significantly without causing hypercalcemia and hypercalciuria across a wide range of replacement doses (6). After his left hemithyroidectomy, the patient then developed HBS with persistent hypocalcemia past postoperative day 4 with a nadir calcium level of 1.87 mmol/L (normal range: 2.12–2.62 mmol/L).

He was treated with intravenous calcium (1000 mg intravenous calcium gluconate on postoperative days 2, 3 and 4). He was concurrently started on oral calcium carbonate 500 mg bid and calcitriol 0.25 μg bid. After improvement of his calcium levels, intravenous calcium was stopped and oral doses were gradually increased to calcium carbonate 2000 mg at lunch and 1000 mg at dinner with calcitriol 0.25 μg bid and vitamin D 10,000 units per week at discharge.

His provoked right leg DVT was treated with anticoagulation for 3 months with an inferior vena cava filter inserted prior to surgical repair of his right intertrochanteric and femoral diaphyseal fracture by orthopedic surgery.

## Outcome and follow-up

**Figure 5:**
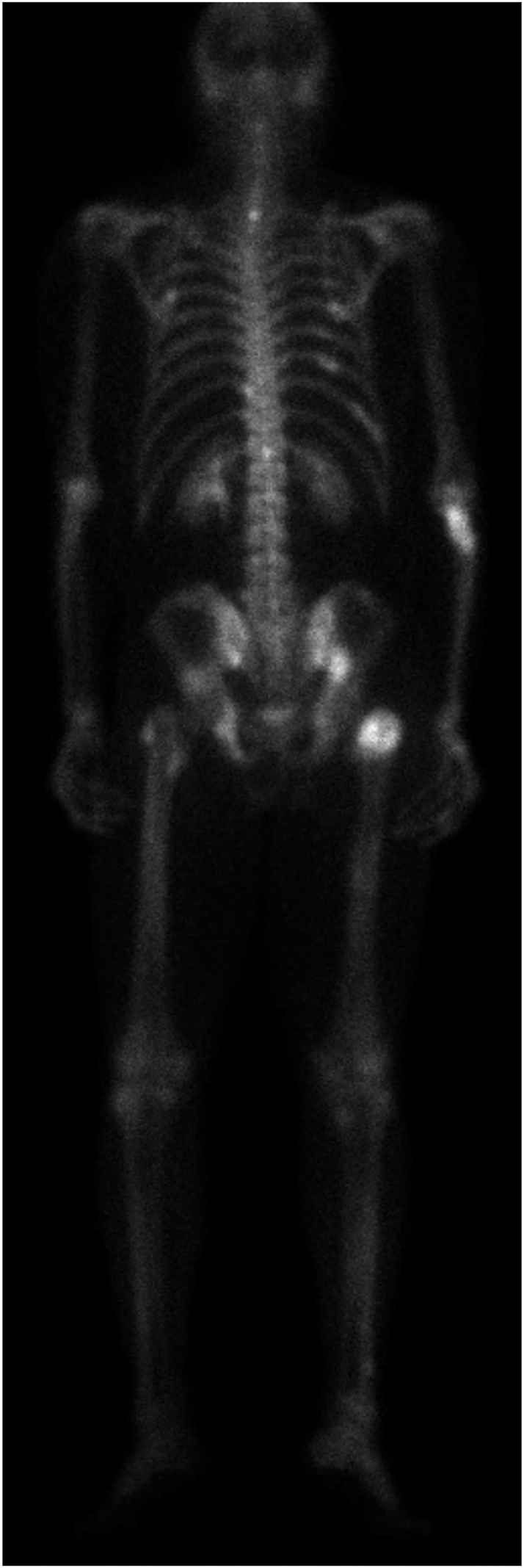
Whole-body bone scan with SPECT/CT of the pelvis. There are numerous foci of moderate to intense activity in the thoracic spine, the rib cage, the right proximal forearm, the pelvis, the proximal femurs and the right proximal tibia. The visualized foci correspond to intramedullary well-circumscribed regions of heterogeneous density, compatible with healing brown tumors (12 months postoperatively).

Several months post-hospitalization, the patient is now walking independently with no symptoms. He required 12 months of calcitriol, calcium and vitamin D supplementation so far and he is being weaned slowly. He now has slight regression of his bone lesions upon bone scan (Fig. 5) and X-rays, with no further pathological fractures or new brown tumors. None of the patient’s tested family members (two children, parents and siblings) had hypercalcemia.

## Discussion

Brown tumors are a rare entity of PHPT with an estimated incidence of 3% in the population diagnosed with PHPT (1). Our case demonstrates several points of interest that differentiate this case from others. PHPT is most common in older women, but our patient was a young man in his 30s. Our patient also presented with multiple brown tumors with two pathological fractures in the long bones. This is particularly rare, as most case reports of PHPT have a single brown tumor. It is thus important to have a multidisciplinary approach with early involvement of orthopedics, otolaryngology and endocrinology. It is also important to ensure appropriate diagnostic work up with a calcium and PTH level test to rapidly identify the underlying etiology. In our case, checking the PTH level avoided a potential delay in diagnosis from the initial presumption that this was a primary bone malignancy or solid organ metastasis.

Another aspect of interest in our case, which differentiates it from other case reports of brown tumors, is the degree of elevation of calcium and PTH, which had the treating team suspecting parathyroid carcinoma, until the tumor was proven to be a benign giant parathyroid adenoma on pathology.

In plain radiographs, brown tumors will appear as well-defined, purely lytic radiolucent lesions that provoke little periosteal reaction. The cortex may be thinned or expanded but will not be penetrated as you may see in metastatic bone disease.

Indications for genetic testing for inheritable parathyroid disease include patients younger than 30 years old with PHPT; those with multigland disease on imaging or history; those with a family history of hypercalcemia or syndromic disease such as MEN1, MEN2A, MEN4 or hyperparathyroidism–jaw tumor syndrome and those with atypical parathyroid adenoma and parathyroid carcinoma (7).

Our patient displayed all the classic risk factors for HBS perioperatively. According to several large case series, around 13% of patients undergoing parathyroidectomy for PHPT develop HBS postoperatively (8).

There is no set PTH level at which HBS is diagnosed. Diagnosis revolves around a profound and persistently low calcium level of less than 2.1 mmol/L for more than 4 days postoperatively, along with hypophosphatemia and normal PTH levels. Hypomagnesemia and hypocalciuria are often associated (9).

A review of the literature has shown that there are specific risk factors associated with a higher likelihood for HBS. Patients with a single adenoma of >2 g developed a higher rate of HBS (68.8%) compared with patients with a single adenoma of <1 g (14.3%) (10). Radiological evidence of bone disease such as brown tumors was also significantly associated with postoperative HBS (9). In addition, elevated preoperative PTH and alkaline phosphatase values indicate a greater chance of developing HBS postoperatively (9). Older age and a high blood urea nitrogen concentration are known risk factors for HBS but were not present in this case.

The pathophysiology behind HBS is not well understood, but it is hypothesized that in patients with preoperative high-risk factors, as previously mentioned, after parathyroidectomy, the normalization of PTH levels provokes a decrease in osteoclastic resorption, with a consequent gain in bone mass. This is believed to be the cause of the rapid and prolonged hypocalcemia, which can last up to a year (8).

Given the rarity of brown tumors, there are no clinical practice guidelines to guide management. Continued follow-up with regular blood tests and imaging to assess for a cure of hyperparathyroidism and healing of bone lesions is of paramount importance. Treating physicians should consider repeating plain radiographs and perhaps other cross-sectional or functional imaging modalities such as bone scans every 6–12 months initially and gradually decrease the frequency of investigations once improvement is documented.

## Declaration of interest

The authors declare that there is no conflict of interest that could be perceived as prejudicing the impartiality of the work.

## Funding

This work did not receive any specific grant from any funding agency in the public, commercial or not-for-profit sector.

## Author contribution statement

J M and V L were the physicians who contributed to the patient’s care. J M drafted the manuscript. W M A assisted with the table, and V L contributed to the reviewing and editing process. M P is the pathologist who was involved in this case, participated in the manuscript and provided the histopathological slides.

## Patient consent

Written informed consent was obtained from the patient for publication of this case report.
